# A Sodium Oxychlorosene-Based Infection Prevention Protocol Safely Decreases Postoperative Wound Infections in Adult Spinal Deformity Surgery

**DOI:** 10.7759/cureus.56109

**Published:** 2024-03-13

**Authors:** Vincent J Alentado, Fezaan A Kazi, Caroline A Potts, Mohamed A Zaazoue, Eric A Potts, Saad A Khairi

**Affiliations:** 1 Neurological Surgery, Goodman Campbell Brain and Spine, Ascension St. Vincent Hospital Indianapolis, Indianapolis, USA; 2 Medicine, Indiana University School of Medicine, Indianapolis, USA; 3 Neurological Surgery, Indiana University School of Medicine, Indianapolis, USA

**Keywords:** complications, spine surgery, oxychlorosene, spinal deformity surgery, infection prevention protocol

## Abstract

Introduction: This study sought to determine the efficacy of a complex multi-institutional sodium oxychlorosene-based infection protocol for decreasing the rate of surgical site infection after instrumented spinal surgery for adult spinal deformity (ASD). Infection prevention protocols have not been previously studied in ASD patients.

Methods: A retrospective analysis was performed of patients who underwent posterior instrumented spinal fusion of the thoracic or lumbar spine for deformity correction between January 1, 2011, and May 31, 2019. The efficacy of a multi-modal infection prevention protocol was examined. The infection prevention bundle consisted of methicillin-resistant *Staphylococcus aureus* testing, chlorhexidine gluconate bathing preoperatively, sodium oxychlorosene rinse, vancomycin powder placement, and surgical drain placement at the time of surgery.

Results: About 254 patients fit the inclusion criteria. Among these patients, nine (3.5%) experienced post-surgical deep-wound infection. Demographics and surgical characteristics amongst infected and non-infected cohorts were similar, although diabetes trended towards being more prevalent in patients who developed a postoperative wound infection (p=0.07). Among 222 patients (87.4%) who achieved a minimum of two years of follow-ups, 184 patients (82.9%) experienced successful fusion, comparing favorably with pseudarthrosis rates in the ASD literature. Rates of pseudarthrosis and proximal junction kyphosis were similar amongst infected and non-infected patients.

Conclusion: An intraoperative comprehensive sodium oxychlorosene-based infection prevention protocol helped to provide a low rate of infection after major deformity correction without negatively impacting other postoperative procedure-related metrics.

## Introduction

Adult spinal deformity (ASD) is a prevalent condition affecting over 60% of patients greater than 60 years of age [1]. ASD is associated with severe pain and reduced quality of life for patients. Posterior instrumented spinal fusion is the current mainstay of treatment for ASD, with large studies demonstrating significant improvements in patient-reported outcomes in a cost-effective manner [2]. Moreover, surgery for ASD demonstrates improved quality of life and cost outcomes for patients compared to nonoperative management [2]. As the population continues to age and the scope of deformity surgery increases, the rate of spinal deformity surgery is rising substantially [3].

Unfortunately, spinal deformity surgery is associated with an increased risk of postoperative wound infection due to long operative times, increased blood loss, multiple osteotomies, and a higher number of fused segments compared to non-deformity spinal surgeries [4,5]. Despite advances in surgical technique, deep wound infections remain prevalent after surgery for ASD [6,7]. Wound infections are associated with multiple hospital admissions, revision surgeries with possible instrumentation revision or removal, lengthy antibiotic treatment, delayed rehabilitation and return to work, and increased costs. Additionally, previous studies have demonstrated high mortality risks associated with postoperative wound infection after spinal surgery [6,8]. Given the increasing amount of ASD surgeries and the negative consequence of postoperative wound infections, decreasing this adverse outcome in ASD surgeries is of paramount importance.

Many previous studies have evaluated the use of infection prevention protocols in lowering rates of surgical site infection in spinal surgery, but few have specifically examined the effects of such a protocol on infection rates after major spinal deformity correction [8,9]. At our institution, prior to protocol implementation, we found a rate of postoperative wound infection of 3.5%. This rate was as high as 5.0% for posterior, instrumented approaches, closely reflecting the infection rate in the literature [6,7]. In this study, we utilized a complex intraoperative sodium oxychlorosene-based infection prevention protocol and evaluated the impact on the rate of postoperative wound infection following ASD surgery. It is important to note that this protocol includes many additional items, such as vancomycin powder, that had been previously utilized at our institution but are now being included in a formalized protocol alongside the novel use of sodium oxychlorosene. As such, the major new component of this infection prevention protocol is sodium oxychlorosene. Sodium oxychlorosene is a hydrochloric acid derivative with a broad spectrum of bactericidal activity, regardless of microorganismal type or resistance. Its use has previously been well-established in general surgery and urology procedures [10-13].

The pH of sodium oxychlorosene is between 6.4 and 6.8, according to the manufacturer, and as such does not cause irritation of open wounds [14]. Furthermore, destruction subsequently leads to the solubilization of microbes due to the release of their protoplasmic contents. Despite its substantial activity against microbes, sodium oxychlorosene demonstrates no effect on solid tissues beyond destroying detached squamous cells, which could lead to infection themselves. Paramount in its efficacy, sodium oxychlorosene demonstrates no systemic absorption and thus no possible allergenicity, allowing for treatment options containing multiple doses with minimal risk of adverse reaction.

Despite the proposed benefits of sodium oxychlorosene on infection prevention, potential delirious effects are not well elucidated within the ASD literature. We deployed sodium oxychlorosene alongside other well-established infection prevention modalities in order to minimize postoperative wound infections in our spinal deformity population. We hypothesized that the inclusion of sodium oxychlorosene irrigation would substantially decrease infection rates compared to those reported in the literature without detriment to rates of postoperative bony union.

## Materials and methods

Infection prevention protocol

In collaboration with our infectious disease department, we developed a multi-institutional infection prevention protocol for spine surgery to improve rates of postoperative wound infection. The main novel component of this protocol for our institution was the inclusion of sodium oxychlorosene. Protocol implementation for spinal deformity patients began on January 1, 2011. The protocol included a preoperative, intraoperative, and postoperative regimen for elective surgical patients. Preoperatively, a medical clearance examination and methicillin-resistant *Staphylococcus aureus* (MRSA) nasal testing were performed. A positive test for MRSA resulted in the patient receiving mupirocin nasal ointment for preoperative decolonization. Additionally, patients were instructed to shower using chlorhexidine gluconate the evening preceding their surgery. Intraoperatively, appropriate antibiotics were administered within one hour before incision and discontinued by 24 hours postoperatively, as per Surgical Care Improvement Project guidelines [15]. Upon completion of instrumentation, and once all positions were checked and the closing process was ready to begin, wound irrigation with one liter of sodium oxychlorosene via pulse lavage was performed. In our initial protocol, a sodium oxychlorosene concentration of 0.2% was utilized for this procedure. However, updated in vitro bactericidal data and institutional infectious disease recommendations in May 2018 caused us to decrease this concentration to 0.05% [16]. After placement, the sodium oxychlorosene was promptly rinsed out with normal saline at the discretion of the operating surgeon. At the start of protocol implementation, normal saline irrigation had been infused with bacitracin, but as per recent U.S. Food and Drug Administration recommendations, we no longer add bacitracin [17]. Two grams of vancomycin powder was next placed in the wound, accompanied by fusion graft. Two surgical drains were then placed and tunneled away from the incision. Drains were kept in place until the drain output from all drains was <150 ml over the preceding 24 hours. In the rare instance of an incidental durotomy, in which drain output usually does not fall to a value of <150 ml/24 hours, the drains were kept for a maximum of seven days. In 2015, we created and implemented an additional surgical checklist to ensure consistency and universal adoption of the protocol amongst all surgeons. The checklist is verbalized by the intraoperative circulating nurse at the point of wound closure to guarantee that irrigation with sodium oxychlorosene, use of vancomycin powder, and placement of drains were deployed appropriately. Such a closure list possesses the added advantage of keying surgeons to key checkpoints in the procedure, such as tightening set screws prior to closing. Since the addition of the checklist, surgeon compliance has been 100%.

Clinical and demographic characteristics

Prior to study initiation, relevant local institutional review board approval was obtained. Ascension St. Vincent Indiana Institutional Review Board issued approval RIN20220022. Electronic medical records (EMRs) were reviewed retrospectively to identify patients who underwent posterior lumbar or thoracolumbar spinal fusions ≥4 levels for an indication of spinal deformity correction at two tertiary care centers between January 1, 2011, and May 31, 2019. Exclusion criteria included age <18 years or infectious etiology for spinal deformity. Revision surgeries that fit the inclusion criteria were included. For all patients, the EMR was retrospectively reviewed to obtain patient indication for surgery, demographic information, type and levels of surgery, and fusion status based on either bony fusion on computed tomography scans or stable fixation on extension/flexion x-rays taken two years postoperatively. Additionally, revision operations for proximal junction kyphosis (PJK) or deep wound infection were documented.

Statistical methods

Research Electronic Data Capture (REDCap) [18] was utilized to ensure that all data were securely collected and stored while the study was conducted. Data analysis was performed by the research team with the assistance of an independent statistician. Categorical variables were analyzed using Fisher’s exact tests, while numerical variables were compared between the cohorts with and without infection using the independent samples t-test. Continuous variables are summarized as means and standard deviations, whereas categorical variables are presented as counts and percentages of the overall cohort. All p values ≤ 0.05 were considered statistically significant.

## Results

Out of the 5047 patients who underwent posterior instrumented spinal surgery at our institutions between January 1, 2011, and May 31, 2019, 254 fit our inclusion criteria. Among these, nine (3.5%) patients experienced postoperative deep wound infection, with one patient dying due to their illness. Demographics and surgical characteristics between the infected and non-infected cohorts were similar (Tables 1, 2).

**Table 1:** Demographic characteristics between non-infected and infected cohorts BMI: Body mass index; ASA: American Society of Anesthesiologists

Demographics	Non-infected cohort (N=245)	Infected cohort (N=9)	p-value
Average age	59.2±15.1	59.0±17.0	1.0
Average BMI	30.2±6.7	32.6±9.0	0.3
Female	161 (65.7%)	7 (77.8%)	0.5
Diabetes	30 (12.2%)	3 (33.3%)	0.07
A1c (%)	6.0±0.8	5.7±0.5	0.3
Smoking history	100 (40.8%)	4 (44.4%)	0.8
ASA grade	2.8±0.5	3.0±0.7	0.2

**Table 2:** Operative characteristics between non-infected and infected cohorts

Operative characteristics	Non-infected cohort (N=245)	Infected cohort (N=9)	p-value
Fusion length	10.3±3.2	10.1±3.4	0.9
Intraoperative temperature	96.9±1.5	97.4±1.5	0.3
Intraoperative transfusion	183 (74.7%)	7 (77.8%)	1.0
Autograft only	25 (10.2%)	1 (11.1%)	1.0
Allograft only	67 (27.3%)	4 (44.4%)	0.3
Combined autograft and allograft	153 (62.4%)	4 (44.4%)	0.3

There was a trend towards a higher rate of diabetic patients in the infected cohort (3/9, 33%) compared to the non-infected cohort (30/245, 12.2%), although this was not statistically significant (p=0.07). Furthermore, preoperative hemoglobin A1C was similar between cohorts.

About 222 (87.4%) patients had a two-year postoperative radiographic follow-up. About 184 (82.9%) of these patients developed successful fusion. Seven patients with a history of infection had two-year follow-up imaging. Two (28.6%) of these patients developed pseudarthrosis compared to 36 (16.7%) in the non-infected cohort (p=0.6, Table 3).

**Table 3:** Two-year radiographic outcomes between non-infected and infected cohorts

Radiographic parameter	Non-infected cohort (N=215)	Infected cohort (N=7)	p-value
Pseudarthrosis	36 (16.7%)	2 (28.6%)	0.6
Proximal junction kyphosis	30 (13.9%)	1 (14.3%)	1.0

PJK rates at two-year follow-ups were also similar amongst cohorts (1/7, 14.3% vs. 30/215, 13/9%, p=1.0).

## Discussion

Postoperative wound infections after ASD surgery worsen the quality of life outcomes for patients. In a retrospective multi-institutional study of prospectively collected data, Haddad et al. [19] investigated 444 ASD patients who underwent instrumented fusion. The authors noted a deep wound infection in 23 (5.2%) patients. About 19 of these patients were matched 3:1 with patients who did not develop a wound infection postoperatively. Oswestry Disability Index (ODI) scores (45.87 vs. 34.22, p=0.05), Short Form-36 (SF-36) physical component scores (31.48 vs. 37.24, p=0.03), and Scoliosis Research Society-22 (SRS-22) Mental (2.77 vs. 3.24, p=0.04) scores were worse at six months in the infected cohort compared to the non-infected cohort. However, at one- and two-year follow-ups, visual analog scale (VAS) back, VAS leg, ODI, SF-36, and SRS-22 scores were similar between cohorts. This demonstrates the short-term negative consequences regarding physical function and mental health after a postoperative wound infection for ASD surgery. While patients with an infection eventually match outcomes compared to their non-infected peers, the lengthy recovery process increases costs and anxiety for patients, while also worsening physical function. Falavigna and associates [20] found that postoperative deep wound infections after lumbar spinal fusion surgery did not negatively impact long-term quality of life outcomes, but patients with a deep wound infection were significantly more likely to be unsatisfied with their surgical results. Despite the negative consequences of postoperative wound infection and the increased risk of wound infection in patients undergoing surgery for ASD, the literature examining infection prevention within this patient population is lacking.

Multiple studies have demonstrated that protocolization is helpful for reducing complication rates in ASD surgery [21-24]. Sethi et al. [21] published their protocol aimed at reducing multiple complications related to ASD surgery. The authors noted a decrease in their infection rates from 8/71 (11.3%) prior to protocol implementation to 1/69 (1.4%) after protocol implementation. Despite this improvement, the protocol did not specifically incorporate infection prevention measures. The authors attributed their improvement in infection rates to more judicious patient selection as opposed to perioperative infection prevention measures. In another study of the benefits of protocolization in ASD surgery, Zeeni et al. [22] investigated their “high-risk” spine protocol incorporating both spinal deformity surgeries as well as patients with significant medical comorbidities. While several preventative measures were implemented to optimize patient results by limiting preventable adverse outcomes, nothing was specifically aimed at infection prevention, and the authors did not discuss infection rates associated with their protocol results.

To our knowledge, there have been no studies investigating an infection prevention protocol in ASD surgeries. In a previous study, we reported the first use of sodium oxychlorosene as an infection-prevention agent in spinal surgery. When investigating all non-infectious etiologies treated with posterior spinal fusion, we found an infection rate of 1.2% following protocol adoption [8]. We noted no adverse outcomes that could be attributed to the use of sodium oxychlorosene or any other component of the protocol. In that study, our overall fusion rate was 89.2%, which is similar to previously reported rates in the literature [25]. In the current study, we aimed to better understand the efficacy of our infection prevention protocol in ASD patients. In a cohort that is at higher risk for both wound infection and non-union, we note an overall infection rate of 3.5% and a bony fusion rate of 82.9%. These findings are similar to those in the reported ASD literature, although rates vary widely [26-31].

Multiple studies have investigated CHG bathing and preoperative MRSA decolonization in spine surgery infection prevention studies with favorable results [8,9,32]. However, they did not investigate spinal deformity surgeries. In contrast, vancomycin powder has been studied as an infection prevention measure in ASD surgery. In a retrospective study of 306 patients, Martin et al. [6] found no difference in infection rates with the use of vancomycin powder (5.3% vs. 5.1%, p=1.0). However, the average number of spinal levels fused was five. In another retrospective study of 215 patients, Theologis et al. [7] found that the addition of vancomycin powder decreased infection rates from 7/64 (10.9%) to 4/151 (2.6%) in their ASD surgical population (p=0.01). In the latter study, the average number of levels fused was 10 and there were no vancomycin-related complications. The authors found that the addition of vancomycin powder resulted in an average direct cost savings of $2444.02 per ASD surgery. The cost of vancomycin powder was $34 at the authors’ institution. The conflicting benefit of vancomycin powder in these two ASD studies may be related to the differences in surgical length and complexity, as observed by the significant differences in construct length between studies. In the current study, we are unable to determine the benefit of any one facet of our infection prevention bundle, but we do note similar infection rates and construct lengths to the treatment arm in the study by Theologis and colleagues [7].

Our initial sodium oxychlorosene concentration of 0.2% was based on a common concentration utilized for bladder irrigation in the treatment of urinary tract infections. The later decision to decrease in utilized concentration was performed at the recommendation of our infectious disease colleagues following their evaluation of the in vitro activity of sodium oxychlorosene against multi-drug resistant organisms. They demonstrated that the minimum inhibitory percentage of sodium oxychlorosene against 161 multi-drug resistant bacteria strains and 28 multi-drug resistant yeast strains was 0.0125% [16]. They recommended a concentration of 400% of this minimum inhibitory concentration. As such, our concentration was decreased to 0.05% to maintain bactericidal activity while minimizing potential side effects. The cost of one liter of sodium oxychlorosene irrigation utilized at our institution is $8.24.

In addition to determining the infection rate with our protocol, we examined complications that have been previously associated with postoperative wound infections in ASD patients. Bony union is paramount to successful outcomes after ASD surgery and postoperative infections have been correlated with pseudarthrosis in this population. This correlation is well elucidated in fracture healing with long bone fractures [33]. The proposed mechanism is related to an exaggerated inflammatory response during osseous infection resulting in the dysregulation of osteoblasts and osteoclasts [34]. In a multicenter retrospective study of 956 ASD patients, Boishardy and associates [35] found that 65 (6.8%) patients developed a postoperative wound infection, and 138 (14.4%) patients failed to obtain bony fusion. Deep wound infection was noted as an independent risk factor for pseudarthrosis (odds ratio=4.4, 95% confidence interval 2.4-7.9). We similarly noted a higher rate of pseudarthrosis in our infection cohort, however this was not statistically significant. It may be that with larger sample sizes of infected patients, our study may have reached statistical significance in regards to pseudarthrosis with postoperative infection, highlighting the importance of infection prevention in this patient population.

Another adverse outcome associated with wound infection in ASD patients is PJK. Haddad et al. [19] found that wound infection significantly increased the rate of PJK (6/19 (31.6%) vs. 5/57 (10.5%), p=0.02). The authors felt that this finding was related to weakened posterior ligamentous tension bands or muscles due to the infection, repeated injury to these structures during revision surgery, or decreased activity leading to muscle atrophy in patients with an infection. In contrast, our study found similar rates of PJK in our infected and non-infected cohorts. As such, further study into this topic is warranted.

Limitations of our study include the retrospective design. Retrospective review of the EMR provided demographic, operative, and clinical outcomes. These data points were also collected from high-volume spine centers at tertiary care institutions. Such characteristics lend the data to being more applicable to similar high-volume hospitals also possessing complex patient populations and the resources and ancillary staff of larger hospitals. This retrospective review is not a case-control study. There was no control group able to be analyzed. Rather, we made a comparison to our institutional infection rates prior to the protocol as well as literature infection rates for similar patient populations with similar indications for surgery. However, bias stemming from the lack of a defined control group cannot be excluded. In addition, the progressive nature of our formalization and adoption of the protocol limits the ability to ascertain the degree to which it was solely responsible for the observed low rate of infection. The initial incomplete adoption of protocol practices prior to checklist introduction may have affected the observed rate of infection and complication rate. Prior to the generalized acceptance of these infection prevention protocols, external validation is necessary.

## Conclusions

ASD surgery carries a higher risk of infection compared to other types of spinal surgeries. The deleterious impact of wound infection on patient outcomes after ASD surgery is significant. This study represents the first investigation of an infection prevention protocol across ASD surgery. At our institution, the implementation of an infection prevention protocol with novel use of sodium oxychlorosene served to provide low infection rates with high safety and efficacy following ASD surgery. Future studies are warranted to further minimize infection rates in this high-risk surgical population.
